# The risk of falling into poverty after developing heart disease: a survival analysis

**DOI:** 10.1186/s12889-016-3240-5

**Published:** 2016-07-15

**Authors:** Emily J. Callander, Deborah J. Schofield

**Affiliations:** Australian Institute of Tropical Health and Medicine, James Cook University, Townsville, Australia; Faculty of Pharmacy, University of Sydney, Sydney, Australia; Discipline of Public Health and Tropical Medicine, Building 41, Douglas Campus, Townsville, QLD 4811 Australia

**Keywords:** Heart disease, Poverty, Longitudinal, Income, Survival analysis, Health economics

## Abstract

**Background:**

Those with a low income are known to have a higher risk of developing heart disease. However, the inverse relationship – falling into income poverty after developing heart disease has not been explored with longitudinal data. This paper aims to determine if those with heart disease have an elevated risk of falling into poverty.

**Methods:**

Survival analysis was conducted using the longitudinal Household Income and Labour Dynamics in Australia survey, between the years 2007 and 2012. The study focused on the Australian population aged 21 years and over in 2007 who were not already in poverty and did not already have heart disease, who were followed from 2007 to 2012. Cox regression models adjusting for age, sex and time-varying co-variates (marital status, home ownership and remoteness of area of residence) were constructed to assess the risk of falling into poverty.

**Results:**

For those aged 20 who developed heart disease, the hazard ratio for falling into income poverty was 9.24 (95 % CI: 8.97–9.51) and for falling into multidimensional poverty the hazard ratio was 14.21 (95 % CI: 13.76–14.68); for those aged 40 the hazard ratio for falling into income poverty was 3.45 (95 % CI: 3.39–3.51) and for multidimensional poverty, 5.20 (95 % CI: 5.11–5.29); and for those aged 60 the hazard ratio for falling into income poverty was 1.29 (95 % CI: 1.28–1.30) and for multidimensional poverty, 1.52 (95 % CI: 1.51–1.54), relative those who never developed heart disease. The risk for both income and multidimensional poverty decreases with age up to the age of 70, over which, those who developed heart disease had a reduced risk of poverty.

**Conclusion:**

For those under the age of 70, developing heart disease is associated with an increased risk of falling into both income poverty and multidimensional poverty.

## Background

Multiple ‘cost of illness’ studies have been produced estimating the indirect costs of cardiovascular disease (CVD): Leal et al estimated that CVD cost €10,768 million in lost productivity due to morbidity in the European Union in 2003, with Germany and the United Kingdom bearing the largest proportion of these costs [1]; the loss of productivity associated with CVD in Australia in 2004 was estimated at €1357 million [2] (converted to Euros for comparison). More recent estimates have been conducted in the United States, with it being projected that the estimated €127,775 million lost in productivity costs in 2010 will increase by 61 % reaching €296,850 million by 2030 (converted to Euros for comparison). However, all these studies use aggregated data and produce aggregated measures, with little attention paid to the actual cost to the individual.

It is known that CVD is significantly associated with lower levels of labour force participation and lower income [3–7]. For example, Schofield *et al* found that people who were out of the labour force because of CVD had a weekly incomes 74 % lower [8]. However, it can be difficult to determine whether CVD or low income came first because these studies are based upon cross-sectional data. The longitudinal studies that have been conducted focus on assessing whether those with a low income have a higher risk of developing CVD. In a Swedish study, it was found that people in the lowest income quartile had a higher risk of developing CVD [9], and similar results have been found in the United States (however this was largely explained by age and gender differences) [10]. To date, no studies have used longitudinal data to assess the inverse relationship: whether those with CVD have a higher of falling into poverty.

The potential influence CVD has on poverty is of great importance due to the sheer number of people affected by CVD. This gives CVD the potential to have a major impact on national poverty rates. It is estimated that one in six Australians had CVD in 2007–08 [11], 84 million American’s suffer from CVD [12], and internationally CVD is the leading cause of death [13]. Furthermore, in many countries the mortality rate for those with CVD is declining [14], reflective of medicine’s successes in treating CVD. This means that a greater number of individual’s must live with the reduced quality of life that is associated with the condition [15]. As such, it may be possible that CVD contributes to a large number of people falling into poverty, which would add a further dimension to the impact the disease has on the living standards of patients, in addition to influencing national poverty trends.

No study to date, has assessed the whether those with cardiovascular disease have an elevated risk of falling into poverty using longitudinal data. This paper seeks to fill this gap, by looking at one aspect of CVD – heart disease – using survival analysis to show the risk people who recently developed heart disease, and survived for the following 5 years, have of falling into income poverty, and also the risk they have of falling into multidimensional poverty. This will be done using a longitudinal dataset that is representative of the Australian population.

## Methods

### Data set sampling and weighting

This study utilised the Household Income and Labour Dynamics in Australia (HILDA) Survey. The HILDA survey is a longitudinal, nationally representative survey of the Australian population. It covers the population living in private dwellings and aged 15 years and over. Wave 1 of the HILDA survey was conducted in 2001. The dataset is available, upon request, from the Australian Department of Human Services.

The survey sampling unit for Wave 1 was the household, with members of private dwellings in Australia making up the reference population. All members of the household are followed over the life of the survey, with detailed individual information recorded for those aged 15 years and over. Household sampling was conducted in a three-stage approach: 1) 488 Census Collection Districts (each containing 200 to 250 households) were selected; 2) 22 to 34 dwellings within each district were then selected; 3) up to three households within each dwelling were selected [16].

The balanced panel of the HILDA survey, which only included respondents who participated in Waves 1 to 12 of the survey, was used for this study. This excluded people who were lost to follow-up. The individuals who participated in each wave were more likely to be ‘female, in older age groups, a member of a couple or divorced, born in Australian or another English speaking country, be non-Indigenous, have a higher level of education attainment, be employed and have a higher skilled job” [16]. To adjust for the potential bias introduced by the loss to follow-up, longitudinal weights were created. The weights aim to produce results that are still nationally representative, despite survey attrition.

The household cross-sectional weights in Wave 1 were derived from the probability of selecting the household. The weights were then calibrated so that the weighted estimates matched benchmark numbers of adults and numbers of children for each state and part of state. The person-level weights were based on the household weights. The person weights were then calibrated to match benchmarks for sex by age, state by part of state, state by labour force status, marital status and household composition. The longitudinal weights were based on the cross-sectional weights of wave 1 and were adjusted for the characteristics of those who were lost to follow-up. To adjust for this attrition, a logistic model calculating the probability of being lost to follow-up based on “age, sex, marital status, ability of speak English, employment status, hours worked, number of children, country of birth, highest level of education, relationship in household, health status, likelihood of moving, number of times moved in last 10 years, whether flagged as reference person for household” and interview characteristics [17]. The adjusted weights were then calibrated back to known “sex by age, state by part of state, state by labour force status, marital status and household composition benchmarks” [17].

### Income, health, education and poverty measures

Two measures of poverty were used in this study: 1) an income poverty measure; and 2) a multidimensional poverty measure. Poverty measures capture the living standards of an individual [18, 19]. Although poverty has traditionally been measured using income-based measures, poverty is now seen as being a multidimensional concept with numerous aspects of a person’s life, not just their income, influencing a person’s poverty status [20, 21].

Income poverty was measured using the total regular income of each household. Household income is composed of ‘regular private income’, which consists of wages/salary, business income, investment income, private pensions, Australian government welfare payments, scholarships, and foreign pensions. This total income was then equivalised using the OECD-modified equivalence scale [22] to adjust for the number and age of household members. Those who had an equivalised income less than 50 % of the median equivalised income for the Australian population of all ages were considered as being in income poverty.

This paper will use the Freedom Poverty Measure to measure multidimensional poverty. The Freedom Poverty Measure [19, 23] was developed specifically for the Australian population. It has been extensively used [23–28], including to assess the multidimensional poverty status of those with CVD (however only cross-sectional data was used in this previous study).

Income, health and education attainment are the key components of the Freedom Poverty Measure. These three factors were selected as they influence participation in an individual’s ability to participate in modern Australian society [19]. Those who are in multidimensional poverty are considered to be in income poverty and have either poor health or a low level of education attainment. The Physical Component Summary (PCS) and Mental Component Summary (MCS) scores from the SF-36 health scale [29] were used to measure health status and identify those who had ‘poor health’. ‘Poor health’ was defied as a PCS or MCS less than 75 % of the average for a person’s age. Education attainment was based upon a person’s highest level of education attainment. A ‘low level of education attainment’ was defined as less than Year 12 (Year 11, Year 10 or below, Certificate I, Certificate II, or certificate undefined) for those aged under 65, or less than Year 9 or lower for those aged over 65 [30–33].

### Measure of heart disease

The HILDA survey was designed to collect data on ‘special topics’ only in certain waves. Detailed information regarding chronic health conditions were only collected in Waves 3, 7 and 9. Waves 3, 7 and 9 of the HILDA survey asked respondent if they had ever been told by a doctor or nurse that they had heart disease. In order to identify people who developed heart disease and their later risk for falling into poverty, data from waves 7 and 9 were utilised. Waves 7 and 9 were chosen (over using data from wave 3) due to the shorter time span between data collection points. Those who stated in Wave 7 and Wave 9 that they had not been told that they have heart disease were considered to have ‘never had heart disease’. Those who stated in Wave 7 that they had not been told that they have heart disease, but in Wave 9 stated that they had been told that they have heart disease, were considered to have ‘developed heart disease between 2007 and 2009’.

### Sample size

**Fig. 1:**
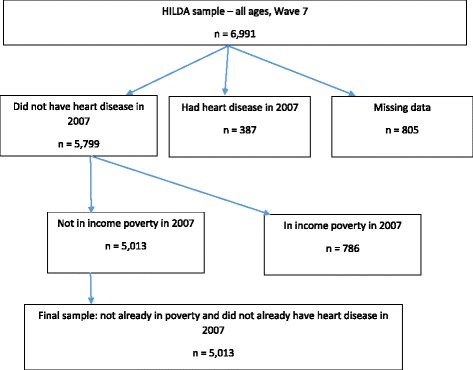
Sample size calculation

The sample in Wave 7 (2007) who were not already in poverty and did not already have heart disease were followed through to Wave 12 (2012). The sample selection is shown in Fig. 1. There were 6991 records on the balanced HILDA dataset. In order to identify those who did not have heart disease in 2007 or 2009, and those who developed heart disease between 2007 and 2009, those with invalid responses (stated they ‘did not know’ or refused the question) to the question ‘Have you ever been told by a doctor or nurse that you have heart disease’ in Wave 7 and Waves 9 were excluded (*n* = 805). Those who had been previously told they had heart disease in Wave 7 were excluded (*n* = 387), and then those who were already in income poverty in Wave 7 were also excluded (*n* = 786). This gave a final sample size of 5013.

### Statistical analysis

The sample was split into two groups: those who developed heart disease between 2007 and 2009, and those who did not develop heart disease. The proportion of people who fell into both income and multidimensional poverty between 2007 and 2012 was then compared for the two groups to determine whether developing heart disease was associated with an elevated risk of falling into income poverty or multidimensional poverty.

Two variables were initially created, one that identified the length of follow-up for respondents, which was terminated by 1) the experience of income poverty or 2) the end of the study; the second identified the censoring variable, whether it was due to the experience of income poverty or whether it was due to the end of the study.

A Kaplan Meier estimate of the survival function was undertaken to explore how the survivor function for those who developed heart disease between 2007 and 2009, and those who had never developed heart disease, changed over time.

Possible confounding variables predictive of survival were then analysed by univariate analysis using chi-square tests. The analysed variables were age, sex, marital status, home ownership, and remoteness of area of residence. A Cox regression model was then computed to show the hazard function for falling into poverty for the two groups. All of the confounding variables were shown to be significant in the univariate analysis, and so all were included in the modelling. All confounding variables were allowed to vary over time. The covariates were tested for proportional hazards assumptions, which held for both models.

The analysis was then repeated using the multidimensional poverty.

Statistical significance was defined as *p* = 0.05. Data processing and analysis were performed with SAS9.4 (SAS Institute, Cary, NC, USA).

## Results

There were 94 records of people aged 21 years and over in 2007 who developed heart disease between 2007 and 2009, and 4934 records of people who had never had heart disease during this time period, who were not already in income poverty in 2007. Once weighted these records represented 140,100 and 7,534,600 people in the population respectively.

The baseline characteristics of the population are shown in Table 1. Of those who had never had heart disease, 49 % were male and the average age in 2007 was 45.9 (SD = 14.2). Of those who developed heart disease between 2007 and 2009, 54 % were male and the average age in 2007 was 60.7 (SD = 12.1). The distribution of the remoteness of area of residence in 2007 was similar between the two groups and a similar proportion were married. However, a larger proportion of those who developed heart disease between 2007 and 2009 owned their own home rather than rented their home.

**Table 1:** Baseline characteristics in 2007

Characteristics	Developed heart disease between 2007 and 2009	Had never had heart disease by 2009
Male	78 700 (54 %)	3 672 000 (49 %)
Age		
21–30	1700 (1 %)	1,182,900 (16 %)
31–40	3700 (3 %)	1,787,300 (24 %)
41–50	24,200 (17 %)	1,842,000 (25 %)
51–60	44,400 (31 %)	1,540,200 (21 %)
61–70	34,200 (24 %)	801,700 (11 %)
71–80	26,600 (19 %)	300,900 (4 %)
81–90	8400 (6 %)	59,900 (1 %)
Married in 2007	104,300 (71 %)	5,441,000 (72 %)
Owns own home in 2007	123,200 (84 %)	5,807,500 (77 %)
Remoteness of area of residence in 2007		
Major city	99,500 (68 %)	5,123,400 (68 %)
Inner regional	33,400 (23 %)	1,626,200 (22 %)
Outer regional	13,200 (9 %)	684,900 (9 %)
Remote	0	100,000 (1 %)

### Income poverty

**Fig. 2:**
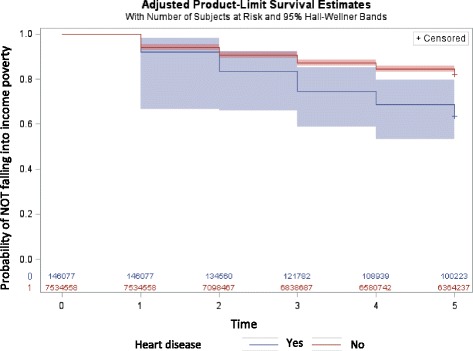
Survival probability for income poverty over time (years) between 2007 and 2012 for those who developed heart disease between 2007 and 2009, and those who never had heart disease

Figure 2 shows the Kaplan Meier survival curve for falling into income poverty for those who developed heart disease between 2007 and 2009, and those who had never had heart disease during this time period. It appears that those who developed heart disease between 2007 and 2009 have a lower survival probability throughout the 5 year study period. This is supported by the log rank chi-squared test of equality of the survivor function (χ^2^ = 14.93, *p* = 0.0001).

**Fig. 3:**
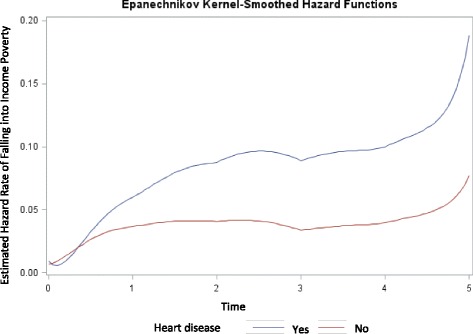
Estimated hazard rate of falling into income poverty between 2007 and 2012

The estimated hazard rate for income poverty is shown in Fig. 3. From this figure we can see that, for those who developed heart disease between 2007 and 2009, the risk for falling into income poverty initially increases rapidly between 2007 and 2009, slightly declines between 2009 and 2011 and then increases again from 2011 to 2012. Whereas for those who never developed heart disease the risk remains somewhat constant up to 2011, then increases from 2011 to 2012.

Results of the univariate analysis show that sex (χ^2^ = 13492.08, *p* < .0001), age (χ^2^ = 876405, *p* < .0001), remoteness of place of residence (χ^2^ = 21278.93, *p* < .0001), marital status (χ^2^ = 107745, *p* < .0001), and home ownership (χ^2^ = 20904.19, *p* < .0001) were all significantly related to falling into income poverty.

The Cox regression model shows that the effect of developing heart disease on the risk of falling into income poverty varies by age (Table 2). The effect of developing heart disease is the strongest for those in younger age groups. The hazard ratio for falling into income poverty for those aged 20 who develop heart disease is 9.24 (95 % CI: 8.97–9.51) and for those aged 30 the hazard ratio is 5.64 (95 % CI: 5.52–5.78), relative to those who never developed heart disease. However, this risk decreases with age up to the age of 70, where those who develop heart disease have a reduced risk of income poverty 0.79 (95 % CI: 0.78–0.80).

**Table 2:** Cox regression model to estimate hazard function of falling into income poverty between 2007 and 2009

Parameter	Parameter estimate	Hazard ratio	*p*-value
Heart disease	3.21	SEE BELOW	<.0001
Age	0.044	SEE BELOW	<.0001
Age*heart disease-never	−0.05	SEE BELOW	<.0001
Male	−0.14	0.87	<.0001
Married	−0.55	0.58	<.0001
Own home	−0.49	0.62	<.0001
Inner regional	0.22	1.24	<.0001
Outer regional	0.31	1.41	<.0001
EFFECT OF 1-UNIT INCREASE IN AGE BY HEART DISEASE STATUS			
	Hazard ratio	95 % CI	
Heart disease –developed between 2007 and 2009	1.00	0.99	1.00
Heart disease–never	1.05	1.05	1.05
EFFECT OF DEVELOPING HEART DISEASE VS NEVER DEVELOPING HEART DISEASE ACCROSS AGES			
	Hazard Ratio	95 % CI	
Age 20	9.24	8.97	9.51
Age 30	5.64	5.52	5.78
Age 40	3.45	3.39	3.51
Age 50	2.11	2.08	2.13
Age 60	1.29	1.28	1.30
Age 70	0.79	0.78	0.80
Age 80	0.48	0.47	0.49
Age 90	0.29	0.29	0.30

Table 2 also shows that being male, married and owning your own home all decrease the hazard ratio for falling into income poverty, but living in inner regional or outer regional areas increases the hazard ratio of falling into poverty.

### Multidimensional poverty

**Fig. 4:**
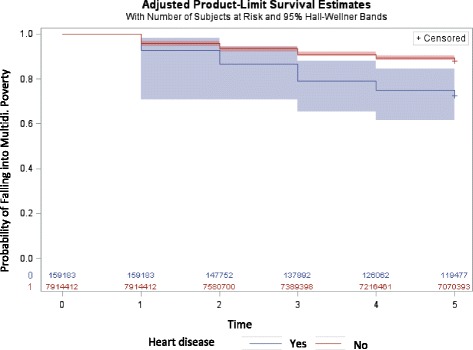
Survival probability for multidimensional poverty over time (years) between 2007 and 2012 for those who developed heart disease between 2007 and 2009, and those who never had heart disease

Figure 4 shows the Kaplan Meier survival curve for falling into multidimensional poverty for those who developed heart disease between 2007 and 2009, and those who had never had heart disease during this time period. This shows that those who developed heart disease between 2007 and 2009 also have a lower survival probability from multidimensional poverty throughout the 5 year study period, and this is also supported by the log rank chi-squared test of equality of the survivor function (χ^2^ = 16.59, *p* < .0001).

**Fig. 5:**
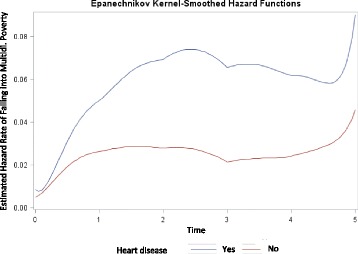
Estimated hazard rate of falling into multidimensional poverty between 2007 and 2012

The estimated hazard rate for multidimensional poverty is shown in Fig. 5. For those who developed heart disease between 2007 and 2009, the risk for falling into multidimensional poverty initially increases rapidly between 2007 and 2010, slightly declines between 2010 and 2011 and then increases again between 2011 and 2012. Whereas for those who never developed heart disease the risk remains somewhat constant up to 2011, then increases from 2011 to 2012.

Results of the univariate analysis show that sex (χ^2^ = 26933.33, *p* < .0001), age (χ^2^ = 701002, *p* < .0001), remoteness of place of residence (χ^2^ = 25432.09, *p* < .0001), marital status (χ^2^ = 100091, *p* < .0001), and home ownership (χ^2^ = 22962.64, *p* < .0001) were all significantly related to falling into multidimensional poverty.

The Cox regression model shows that the effect of developing heart disease on the risk of falling into multidimensional poverty varies by age (Table 3). The effect of developing heart disease is again the strongest for those in younger age groups. The hazard ratio for falling into multidimensional poverty for those aged 20 who develop heart disease is 14.21 (95 % CI: 13.76–14.68), and for those aged 30 the hazard ratio is 8.13 (95 % CI: 7.93–8.34), relative to those who never developed heart disease. However, again this risk decreases with age up to the age of 70, where those who develop heart disease have a reduced risk of income poverty 0.87 (95 % CI: 0.86–0.90).

**Table 3:** Cox regression model to estimate hazard function of falling into multidimensional poverty between 2007 and 2009

Parameter	Parameter estimate	Hazard ratio	*p*-value
Heart disease – never	3.77	See below	<.0001
Age	0.04	See below	<.0001
Age*heart disease-never	−0.06	See below	<.0001
Male	−0.24	0.79	<.0001
Married	−0.55	0.57	<.0001
Own home	−0.58	0.56	<.0001
Inner regional	0.34	1.41	<.0001
Outer regional	0.20	1.22	<.0001
EFFECT OF 1-UNIT INCREASE IN AGE BY HEART DISEASE STATUS			
	Hazard ratio	95 % CI	
Heart disease –developed between 2007 and 2009	0.99	0.99	0.99
Heart disease–never	1.05	1.05	1.05
EFFECT OF DEVELOPING HEART DISEASE VS NEVER DEVELOPING HEART DISEASE ACCROSS AGES			
	Hazard Ratio	95 % CI	
Age 20	14.21	13.76	14.68
Age 30	8.13	7.93	8.34
Age 40	4.65	4.56	4.74
Age 50	2.66	2.63	2.70
Age 60	1.52	1.51	1.54
Age 70	0.87	0.86	0.88
Age 80	0.50	0.49	0.51
Age 90	0.29	0.28	0.29

## Discussion

Developing heart disease increases the risk of falling into both income poverty and multidimensional poverty, amongst those under 70 years of age. Between 2007 and 2012, 31 % of those who developed heart disease fell into income poverty and 25 % fell into multidimensional poverty, by comparison only 15 % of people who did not develop heart disease fell into income poverty and only 11 % fell into multidimensional poverty. Previous studies using cross sectional data have found that those who have CVD have a greater chance of being in poverty [23, 34] and some longitudinal studies have shown that those with a low income or those who are unemployed have an elevated risk of developing CVD [9, 10]. However, this is the first study to look at the inverse relationship, with the results clearly demonstrating that heart disease elevates the risk of falling into both multidimensional poverty and income poverty for those under the age of 70.

Given the high prevalence of heart disease within developed countries, and the high risk those with heart disease have of falling into poverty, heart disease should be seen as a major driver of national poverty rates. It thus warrants the attention of social security departments, in addition to health departments. This study has shown that those in their 20s who develop heart disease have over 10 times the risk of falling into poverty, while the magnitude of this risk does decline with increasing age, even those aged in their 50s who develop heart disease still have over twice the risk of falling into poverty. While traditionally heart disease has been a condition associated with the elderly, relatively young people are still affected. In Australia it is estimated that 6 % of 25 to 34 year olds, 11 % of 35 to 44 year olds, 20 % of 45 to 54 year olds and 35 % of 55 to 64 year olds suffer from a circulatory system condition [35], which is similar to the rates reported in the United States of more than 10 % of 20 to 39 year olds and more than 35 % of 40 to 59 year olds [12].

The results show that the risk for falling into poverty is highest in the 1 to 3 years following diagnosis, which may be explained by patients taking time out of the workforce to seek medical treatment. This time out of the workforce may be compensated for with social security or welfare payments. Within Australia people who have medical confirmation that they cannot work due to a health condition and meet the eligibility criteria are eligible for welfare payments, with the United Kingdom and the United States offering similar arrangements [36–38]. However, the value of social security payments is relative low and should be viewed as a safety net only [39]. Social security systems should focus upon keeping people who survive heart disease in employment or helping them re-join the workforce, and also allowing ease of access to payments as people may be in and out of employment while they are seeking treatment.

This paper does have limitations that need to be acknowledged. The study was only able to focus upon those with heart disease, and did not include other conditions of the circulatory system, due to low sample numbers within the HILDA survey. Furthermore, the study relies on self-reported health status, and assumes the people are accurately able to recall whether or not they have been told by a doctor or nurse that they had heart disease and the income measure is also based upon self-reported data and so is subject to individuals being able to accurately identify their current income. However, self-reported health data is seen to be a valid measure [40] of health status, and self-reported income is also a standard measure, thus the potential for any bias was considered by the authors to be minimal.

## Conclusions

In spite of these limitations, this paper has been able to identify heart disease as a risk factor for both income poverty and multidimensional poverty for people under the age of 70. To date the few longitudinal studies that have been conducted exploring the relationship between cardiovascular diseases and income have only looked at low income as a risk factor for developing CVD. To the authors best knowledge this is the first study to document the inverse relationship, although the findings were limited to those with heart disease. These findings are significant given the high prevalence of heart disease amongst the population of developed nations.

## Abbreviations

CI, confidence interval; CVD, cardiovascular disease; HILDA, Household Income and Labour Dynamics in Australia Survey; MCS, Mental Component Summary; OECD, Organisation for Economic Co-operation and Development; PCS, Physical Component Summary; SD, standard deviation
